# The effect of sex on social cognition and functioning in schizophrenia

**DOI:** 10.1038/s41537-021-00188-7

**Published:** 2021-12-01

**Authors:** Marta Ferrer-Quintero, Michael F. Green, William P. Horan, David L. Penn, Robert S. Kern, Junghee Lee

**Affiliations:** 1 Parc Sanitari Sant Joan de Déu, Sant Boi de Llobregat Barcelona, Spain; 2 Department of Social Psychology and Quantitative Psychology, University of Barcelona, Barcelona, Spain; 3 Centro de Investigación Biomédica en Red (CIBERSAM), Madrid, Spain; 4 Department of Psychiatry and Biobehavioral Sciences, University of California Los Angeles, Los Angeles, CA USA; 5 Desert Pacific Mental Illness Research, Education, and Clinical Center, Greater Los Angeles Veterans Affairs Healthcare System, Los Angeles, CA USA; 6 VeraSci, Durham, NC USA; 7 Department of Psychology and Neuroscience, University of North Carolina-Chapel Hill, Chapel Hill, NC USA; 8 School of Behavioural and Health Sciences, Australian Catholic University, Sydney, Australia; 9 Department of Psychiatry and Behavioral Neurobiology, The University of Alabama at Birmingham, Birmingham, AL USA; 10 Comprehensive Neuroscience Center, The University of Alabama at Birmingham, Birmingham, AL USA

**Keywords:** Schizophrenia, Human behaviour

## Abstract

Social cognitive impairment is a core feature of schizophrenia and plays a critical role in poor community functioning in the disorder. However, our understanding of the relationship between key biological variables and social cognitive impairment in schizophrenia is limited. This study examined the effect of sex on the levels of social cognitive impairment and the relationship between social cognitive impairment and social functioning in schizophrenia. Two hundred forty-eight patients with schizophrenia (61 female) and 87 healthy controls (31 female) completed five objective measures and one subjective measure of social cognition. The objective measures included the Facial Affect Identification, Emotion in Biological Motion, Self-Referential Memory, MSCEIT Branch 4, and Empathic Accuracy tasks. The subjective measure was the Interpersonal Reactivity Index (IRI), which includes four subscales. Patients completed measures of social and non-social functional capacity and community functioning. For objective social cognitive tasks, we found a significant sex difference only on one measure, the MSCEIT Branch 4, which in both patient and control groups, females performed better than males. Regarding the IRI, females endorsed higher empathy-related items on one subscale. The moderating role of sex was found only for the association between objective social cognition and non-social functional capacity. The relationship was stronger in male patients than female patients. In this study, we found minimal evidence of a sex effect on social cognition in schizophrenia across subjective and objective measures. Sex does not appear to moderate the association between social cognition and functioning in schizophrenia.

## Introduction

Social cognitive impairment is a core feature of schizophrenia. During the past two decades, a large body of work has shown the pervasive nature of social cognitive impairment and its critical role in poor functioning in schizophrenia^1–3^. However, surprisingly little is known about whether key biological variables, such as sex, moderate the level of social cognitive impairment and the strength of the association between social cognitive impairment and community functioning in schizophrenia.

While less is known about sex difference in social cognition in schizophrenia, several studies have shown the effect of sex on other core features of schizophrenia. For instance, female patients with schizophrenia tend to have older age of onset^4,5^, better premorbid functioning^4^, and better social functioning^6,7^. Better social functioning of female patients raises an interesting question as to whether any key determinants of functioning, such as non-social cognition and social cognition, may also differ between male and female patients. For non-social cognition, studies on sex differences in schizophrenia have produced mixed findings, such that some found better performance in female than male patients^8–10^, whereas others found the opposite^11,12^ or no difference between female and male patients^13–15^. As these studies focused on different domains of non-social cognition, it is possible that sex differences in cognition in schizophrenia may vary across non-social cognitive domains. The inconsistent findings of these studies raise a possibility that sex differences in social cognition in schizophrenia may differ depending on the type of measures (e.g., subjective versus objective measures), which in turn may affect the relationships between social cognition and functioning.

There is a pervasive impression that compared to males, females are generally better at processing social information, including emotional expressions. Several studies have empirically examined this possibility in healthy populations using both subjective and objective social cognitive measures across multiple domains of social cognition. For subjective social cognitive measures, on which participants self-reported their social cognitive abilities, females reported higher empathy^16^ and higher emotional intelligence^17^, compared to males. Studies with objective social cognitive tasks present a more nuanced pattern of female advantage in processing social stimuli. A majority of studies on sex differences examined emotion identification or emotion discrimination using face stimuli. Several studies failed to find sex differences^17,18^, while some found slightly better performance in females for emotion recognition, especially for negative emotions^19–21^. Similarly, when discriminating emotional body movement of point-light walkers, females performed slightly better at discriminating emotional body movement of point-light walkers^22^ or comparably to males^23^. Females performed slightly better at understanding thoughts of another person^24^ or the emotional state of another person^25^ compared to males. Thus, it appears that sex differences in healthy samples are more consistently found using subjective versus objective social cognitive measures.

Several studies examined sex differences in social cognition in schizophrenia using objective social cognitive tasks. Female and male patients showed comparable performance when recognizing facial emotions^26–28^ or understanding the thoughts of another person (i.e., mental state attribution)^26–28^. While these findings suggest a lack of sex difference in schizophrenia, most studies employed only objective social cognitive tasks and primarily focused on perception of emotional expressions or mental state attribution. Thus, it remains to be determined whether sex differences in schizophrenia exist for subjective social cognition or whether sex differences are present for other social cognitive domains beyond emotion perception and mental state attribution.

To examine sex differences in social cognition in schizophrenia, this study presents a secondary analysis of data from a two-site case-control study, Social Cognition and Functioning in Schizophrenia (SCAF)^29,30^. Specifically, by adapting paradigms from social cognitive and affective neuroscience, the SCAF project assessed several social cognitive domains that have not been previously examined in schizophrenia, including self-referential memory and empathic accuracy. The SCAF project also included a subjective social cognitive measure of empathy. Thus, this data set is well suited to examine the following research questions: (1) whether there are sex differences in the levels of social cognitive performance of schizophrenia patients and (2) whether sex moderates the associations between social cognition and functioning in schizophrenia.

## Results

### Demographic and clinical characteristics

Table 1 shows the demographical and clinical characteristics of the sample separated by sex. For age and parental education, we did not find any significant effect. For personal education and MCCB Neurocognitive Composite Score, we only found significant group effects. Within the schizophrenia group, we found a significant sex effect on SANS total (*F*_(1,244)_ = 63.49, *p* < 0.05, *η*^2^_p_ = 0.025), but not on age of onset and BPRS total. Female patients with schizophrenia showed lower levels of negative symptoms assessed with SANS compared to male patients with schizophrenia. For functional capacity and community functioning, we found a significant sex effect on MASC total (*F*_(1,236)_ = 9.42, *p* < 0.01, *η*^2^_p_ = 0.038) and on RFS total (*F*_(1,331)_ = 8.20, *p* < 0.01, *η*^2^_p_ = 0.024), but not on UPSA total. Female patients with schizophrenia showed higher levels of functional capacity on social domain and better community functioning compared to male patients with schizophrenia.

**Table 1:** Demographic and clinical characteristics.

	Patients		Controls	
	Female (*N* = 61)	Male (*N* = 187)	Female (*N* = 31)	Male (*N* = 56)
Age	42.4 (12.4)	42.1 (12.4)	42.3 (9.6)	42.7 (10.4)
Personal Education (yrs)^a^	12.7 (1.8)	12.5 (1.7)	14.7 (1.9)	14.7 (1.9)
Parental Education (yrs)	13.8 (2.9)	13.5 (3.1)	13.4 (2.6)	13.3 (2.8)
*Ethnicity*				
Hispanic	5	16	3	6
Not Hispanic	56	171	28	50
*Race*				
Asian	1	6	1	2
Hawaiian/other Pacific Islander	0	1	1	0
Black	29	73	10	15
White	30	99	18	38
More than one race	1	8	1	1
Age of onset (yrs)	22.4 (9.9)	21.1 (5.9)		
SANS^b^	7.0 (3.1)	8.1 (3.2)		
BPRS	45.9 (13.3)	45.1 (13.8)		
UPSA^b^	0.77 (0.12)	0.72 (0.13)		
MASC	3.68 (0.46)	3.46 (0.49)		
RFS^b^	18.1 (5.3)	17.2 (4.5)		
MCCB neurocognitive composite^c^	33.5 (13.0)	29.8 (12.7)	47.7 (12.6)	45.9 (12.1)

Values are given as mean (standard deviation).

*SANS* the Scale for the Assessment of Negative Symptoms, *BPRS* the Brief Psychiatric Rating Scale-24 item, *UPSA* the University of California at San Diego Performance-based Assessment, *MASC* the Maryland Assessment of Social Competence, *RFS* the Role Functioning Scale, *MCCB* MATRICS Cognitive Consensus Battery.

^a^A significant effect of group (*F*_(1,331)_ = 75.11, *p* < 0.001, *η*^2^_p_ = 0.185) indicating that patients had lower levels of personal education than controls.

^b^Significant sex difference within the patient group.

^c^A significant effect of group (*F*_(1,326)_ = 84.44, *p* < 0.001, *η*^2^_p_ = 0.206) indicating that patients showed poorer performance than controls.

### Objective and subjective social cognitive tasks

**Fig. 1:**
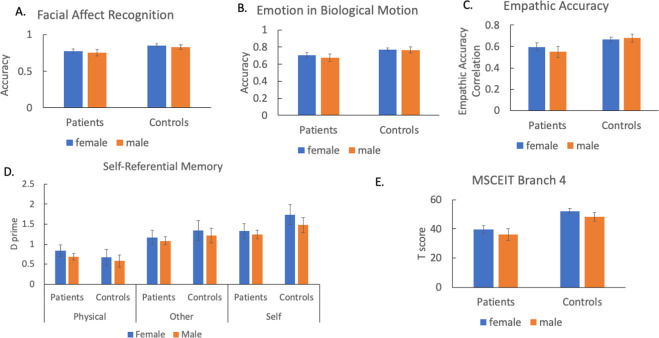
Performance of patients and controls on objective social cognitive tasks. **A** Facial affect recognition, **B** Emotion in biological motion, **C** Empathic accuracy, **D** Self-referential memory, and **E** MSCEIT branch 4. Error bars indicate 95% confidence interval. *MSCEIT* the Mayer–Salovey–Caruso Emotional Intelligence Test 2.0.

**Fig. 2:**
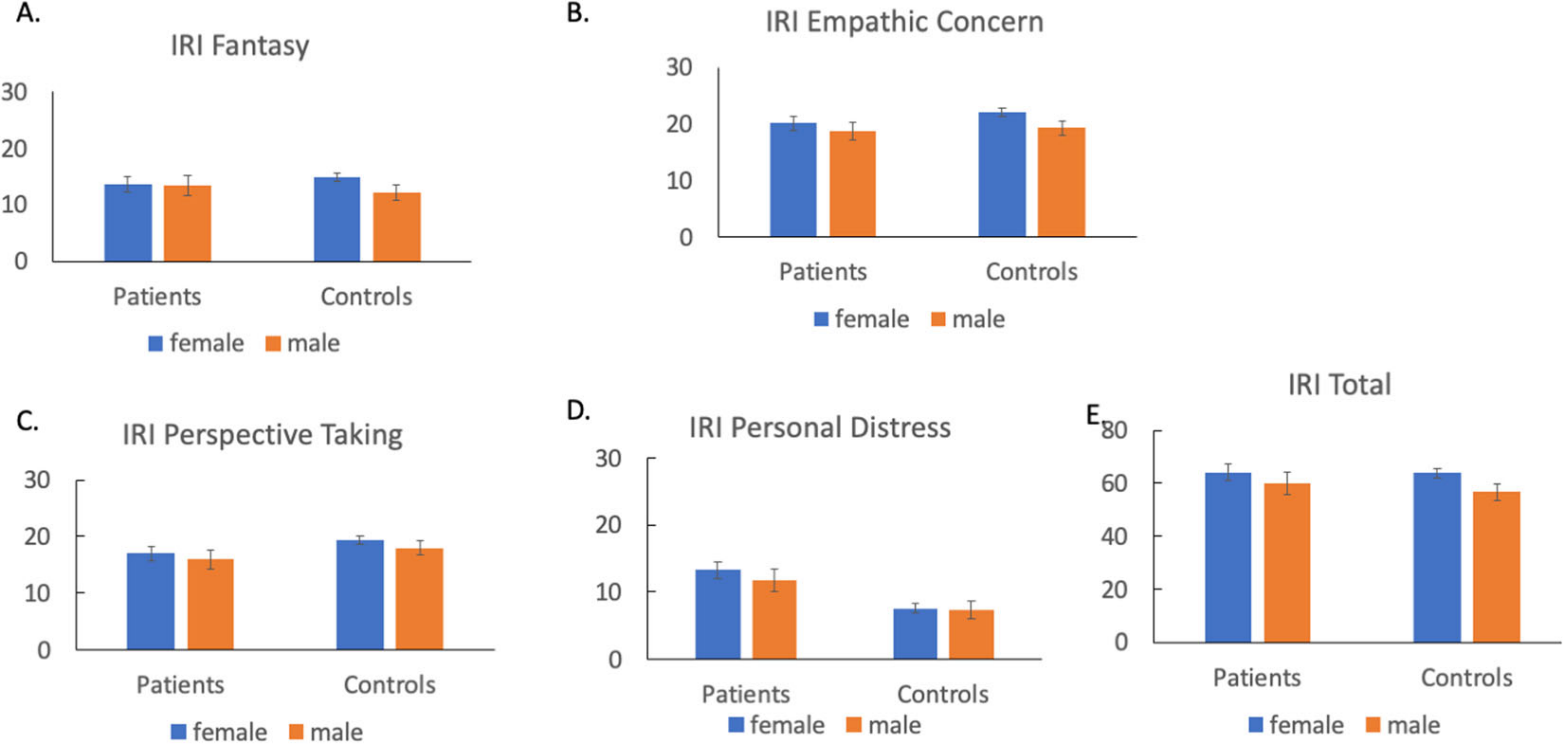
Performance of patients and controls on the subjective social cognitive measure. **A** IRI fantasy, **B** IRI empathic concern, **C** IRI perspective taking, **D** IRI personal distress, and **E** IRI Total. Error bars indicate 95% confidence interval. *IRI* the Interpersonal Responsivity Index.

Figures 1 and 2 show performance of patients and controls on objective and subjective cognitive tasks, respectively. Tables 2 and 3 show statistics from two-way ANOVAs and a repeated measures ANOVA. For Facial Affect Recognition task, Emotion in Biological Motion task and Empathic Accuracy task, we did not find any significant effect involving sex. Similarly, no significant main effect of sex or significant interaction involving sex was found for the Self-Referential Memory task. For the MSCEIT Branch 4, we found a significant effect of sex such that female participants performed better than male participants, and this sex effect did not differ between patients and controls as evidenced by a non-significant sex by group interaction.

**Table 2:** Performance on objective social cognitive measures.

	Inferential statistics	*P* value	Effect size (*η*^2^_p)_	95% confidence interval of parameter estimates^a^
*Facial affect recognition*^b^				
Group	*F*_(1,329)_ = 20.14	<0.001	0.06	[−0.11, −0.04]
Sex	*F*_(1,329)_ = 1.88	NS	0.01	
Group by sex	*F*_(1,329)_ = 0.001	NS	0.00	
*Emotion in biological motion*^b^				
Group	*F*_(1,323)_ = 21.76	<0.001	0.06	[−0.121, −0.05]
Sex	*F*_(1,323)_ = 1.19	NS	0.00	
Group by sex	*F*_(1,323)_ = 0.57	NS	0.00	
*Self-referential memory*				
Group	*F*_(1,325)_ = 1.98	NS	0.01	
Sex	*F*_(1,325)_ = 2.82	NS	0.01	
Group by sex	*F*_(1,325)_ = 0.10	NS	0.00	
Condition	*F*_(2,650)_ = 227.04	<0.001	0.41	
Condition by group	*F*_(2,650)_ = 21.08	<0.001	0.06	
Condition by sex	*F*_(2,650)_ = 0.46	NS	0.00	
Condition by sex by group	*F*_(2,650)_ = 1.41	NS	0.00	
*Empathic accuracy*^b^				
Group	*F*_(1,316)_ = 24.13	<0.001	0.07	[−0.17, −0.08]
Sex	*F*_(1,316)_ = 0.71	NS	0.00	
Group by sex	*F*_(1,316)_ = 2.15	NS	0.01	
*MSCEIT branch 4*^b,c^				
Group	*F*_(1,326)_ = 69.03	<0.001	0.18	[−15.72, −8.96]
Sex	*F*_(1,326)_ = 5.83	<0.05	0.02	[−1.08, 8.82]
Group by sex	*F*_(1,326)_ = 0.02	NS	0.00	

*MSCEIT* the Mayer–Salovey–Caruso Emotional Intelligence Test 2.0.

^a^A 95% confidence interval for the parameter estimate is reported for significant group or sex effects.

^b^Females performed better than controls.

^c^Patients performed worse than controls.

**Table 3:** Performance on subjective social cognitive measures.

	Inferential statistics	*P* value	Effect size (*η*^2^_p)_	95% confidence interval of parameter estimates^a^
*IRI fantasy*				
Group	*F*_(1,329)_ = 0.00	NS	0.00	
Sex	*F*_(1,329)_ = 4.46	NS	0.01	
Group by sex	*F*_(1,329)_ = 3.25	NS	0.01	
*IRI empathic concern*^b^				
Group	*F*_(1,329)_ = 4.18	NS	0.01	
Sex	*F*_(1,329)_ = 11.43	<0.01	0.03	[0.79, 4.79]
Group by sex	*F*_(1,329)_ = 1.42	NS	0.00	
*IRI perspective taking*^c^				
Group	*F*_(1,329)_ = 11.32	<0.01	0.03	[−3.45, −0.56]
Sex	*F*_(1,329)_ = 3.32	NS	0.01	
Group by sex	*F*_(1,329)_ =0.73	NS	0.00	
*IRI personal distress*^c^				
Group	*F*_(1,329)_ = 61.78	<0.001	0.16	[2.97, 5.86]
Sex	*F*_(1,329)_ = 1.98	NS	0.01	
Group by sex	*F*_(1,329)_ = 1.20	NS	0.00	
*IRI total*^b^				
Group	*F*_(1,329)_ = 1.03	NS	0.00	
Sex	*F*_(1,329)_ = 11.58	<0.01	0.03	[1.65, 12.42]
Group by Sex	*F*_(1,329)_ = 0.77	NS	0.00	

*IRI* the interpersonal reactivity index.

^a^A 95% confidence interval for the parameter estimate is reported for significant group or sex effects.

^b^Patients performed worse than controls.

^c^Females performed better than controls.

For the IRI, on Empathic Concern and Fantasy subscale, we found a significant sex effect, but no interaction between sex and group. Female participants reported significantly higher scores on Empathic Concern, and this pattern did not differ between patients and controls. On the Fantasy subscale, a sex effect was no longer significant after correcting for multiple comparisons. On the Perspective Taking and Personal Distress subscales, no effect involving sex was significant. Finally, for IRI total score, we found a significant effect of sex after correcting for multiple comparisons, but no group by sex interaction. Across both patient and control groups, females had higher IRI total scores.

### The moderating role of sex in relationships between social cognition and functioning

Table 4 presents findings from linear regression analyses that examined the moderating role of sex in relationships between social cognition and functioning. As the findings did not change when negative symptoms assessed with SANS, neurocognition assessed with MCCB, age, or site were included in the model, we present findings without these covariates below. Bivariate correlations among variables in male and female patients are presented in the Supplement.

**Table 4:** Linear multiple regression analyses to examine the moderating role of sex in associations between social cognition and functioning.

		Step 1		Step 2^a^			Step 3^b^				
		*R*^2^	AIC	*R*^2^	Δ*R*^2^	AIC	*R*^2^	Δ*R*^2^	AIC	Unstandardized coefficients^c^	
										Female	Male
Social cognitive composite	UPSA	0.343**	−1075	0.345**	0.002	−1074	0.363**	0.018*	−1079	0.05**	0.093**
	MASC	0.071**	−379	0.097**	0.026*	−384	0.098**	0.001	−382		
	RFS	0.079**	1058	0.080**	0.001	1057	0.081**	0.001	1059		
IRI total	UPSA	0.000	−971	0.013	0.013	−972	0.013	0.000	−970		
	MASC	0.023**	−362	0.055**	0.032**	−368	0.055**	0.000	−366		
	RFS	0.002	1125	0.026	0.024**	1118	0.026	0.000	1120		

*AIC* Akaike Information Criterion, *UPSA* the University of California at San Diego Performance-based Assessment, *MASC* the Maryland Assessment of Social Competence, *RFS* the Role Functioning Scale, *IRI* the Interpersonal Reactivity Index.

*p < 0.05; **p < 0.01.

^a^Step 2 included sex as a dummy variable.

^b^Step 3 included interaction between sex and predictors.

^c^For significant interactions, unstandardized coefficients are presented. The significance of unstandardized coefficients was examined using *t*-tests.

With a social cognitive composite score as a predictor and UPSA as a predicted variable, we found a significant interaction between sex and social cognition such that the relationship between a social cognitive composite and UPSA was stronger in male than in female patients. For the MASC, we observed a significant effect of social cognition and a significant effect of sex, but no significant interaction. Sex did not moderate the association between objective social cognition and MASC. For RFS, we observed only a significant effect of social cognition.

Regarding the relationships between subjective social cognition and functioning, we found a significant effect of the IRI total and a significant effect of sex, but no interaction between IRI total and sex when predicting MASC. The strength of association between the IRI total and MASC did not differ between female and male patients. For UPSA and RFS, we did not find any significant effect.

## Discussion

This study examined the effect of sex on the levels of social cognitive impairment and the relationship between social cognition and functioning (functional capacity and functional outcome). Overall, the findings of this study do not strongly support a female advantage for social cognitive ability. For objective social cognitive tasks, we found a significant sex effect, but no sex by group interaction on the MSCEIT Branch 4, a measure of emotional regulation. Females performed better than males, and this effect was similar across patients and controls. A sex effect was not found on other objective social cognitive measures. Regarding subjective social cognition in both patient and control groups, females reported greater empathic concern than males. We did not find any sex differences on other subscales of the IRI. Finally, we found that sex moderated the association between objective social cognition and non-social functional capacity. This relationship between objective social cognition and functional capacity was stronger in male than female patients. However, sex did not moderate the relationships between objective social cognition and other measures of functioning. Nor did sex moderate the relationship between subjective social cognition and functioning in schizophrenia.

In this study, female patients showed less severe negative symptoms, better functional capacity in the social domain, and better community functioning than male patients. These findings add to the existing literature on sex differences in schizophrenia^6,7^, suggesting that the course of illness differs between female and male schizophrenia patients. In this context, it is notable that we did not find strong evidence on sex difference in social cognition, a key determinant of poor functioning in schizophrenia. The lack of sex effect is consistent with recent studies showing comparable performance between female and male patients on social cognitive tasks^15,27^. Further, our regression analyses showed that sex moderated the relationship between objective social cognition and UPSA, but not other measures of functioning. Overall, the role of social cognition in community functioning in schizophrenia does not seem to differ much between female and male patients, suggesting that any intervention for improving social cognition is likely to have similar effects in both female and male patients.

For objective social cognitive measures across both patients and controls, females performed better than males on a measure of emotion regulation, consistent with a previous study^25^. However, across both groups, females and males performed similarly on the measures of emotion identification, emotional biological motion and empathic accuracy. This is consistent with previous studies in healthy individuals that showed the lack of sex differences in emotion identification^17,18^ and emotional biological motion perception^23^. Thus, it appears that females and males recognize or infer emotional social cues in a similar way but diverge when asked to regulate emotional responses in a social situation. As this study did not include any measures at a neural level, the question remains as to whether this pattern of sex differences across emotional domains exists at the neural level. Whereas other objective measures on emotional processing that this study employed primarily relied on visual stimuli or video clips, the MSCEIT Branch 4 used vignettes of social situations that required participants to rely on a language processing ability. It remains to be determined whether females and males perform differently on social cognitive tasks with greater demand on language processing. Beyond emotional processing, this study also found that females and males performed in a comparable way on the measure of self-referential memory. This is consistent with a recent neuroimaging study^31^ in which females and males showed a similar pattern of neural activations related to self-referential processing.

Similar to objective social cognitive measures, we found sex differences on the IRI Empathic Concern subscale, but not on other subscales. The Empathic Concern subscale involves one’s emotional responses to others (e.g., feeling compassion). The Personal Distress subscale concerns one’s own feelings of anxiety or distress in social situations, and the Perspective Taking subscale asks one’s tendency to take another’s perspective in social situations. Taken together, our findings from the subjective social cognitive measure suggest that females may endorse greater emotional responses, such as sympathy or compassion toward others, but these greater emotional responses to others do not result in greater distress or anxiety. It is possible that the greater emotional regulation of females we observed with the objective social cognitive task may play a role in modulating one’s own emotional feeling in the presence of greater emotional reactivity to others.

The findings of this study also raise a question as to what factors other than social cognition may be related to better community functioning in female patients. For example, higher cognitive reserve has been implicated in better social functioning in schizophrenia^32^. It is possible that female patients may have higher cognitive reserve. Schizophrenia patients tend to overestimate their ability to accurately perform on social cognitive tasks^33^, which was related to poorer community functioning in schizophrenia^34^. It will be important to carefully examine whether these variables may differentially affect community functioning in female compared to male patients.

Our study had several limitations. The study included chronic patients, so it remains to be determined whether a similar pattern of sex differences is observed in patients with recent-onset psychosis or in individuals at risk for developing psychosis. Similarly, as this study employed behavioral measures, it needs to be examined whether a similar pattern of sex differences in social cognition in schizophrenia is present at a neural level. This study only included one measure of subjective social cognition, so it will be important to examine whether sex differences can be observed on other domains of social cognition assessed with subjective measures. Finally, as the study sample was not balanced on sex, it will be important to replicate these findings using a more balanced sample that also better represents the general population of patients.

In summary, this two-site case-control study used a large battery of measures across multiple social cognitive domains to examine the effect of sex on the levels of social cognitive impairment and the relationship between social cognitive impairment and functioning in schizophrenia. Our findings suggest that the sex difference in social cognition in schizophrenia is not strong and may vary from domain to domain. Sex moderated the relationship between objective social cognition and non-social functional capacity, but not other measures of functioning. Our finding of sex not moderating the relationship between social cognition and community functioning in schizophrenia also suggests that social cognition is less likely to explain better community functioning of female versus male patients with schizophrenia.

## Methods

### Participants

This study included 248 patients with schizophrenia and 87 healthy controls from two sites: (1) University of California, Los Angeles (UCLA)—outpatient treatment facilities in the Los Angeles area and mental health clinics at the VA Greater Los Angeles Healthcare System (VAGLAHS) and (2) University of North Carolina (UNC)—Chapel Hill Schizophrenia Treatment and Evaluation Program and community mental health clinics in the Chapel Hill area. Healthy controls were recruited through internet advertisements. All participants provided written informed consents after procedures were fully explained, as approved by the Institutional Review Boards at University of California Los Angeles, VAGLAHS, and UNC.

Selection criteria have been described elsewhere^29^. Briefly, for patients they included: (1) Diagnostic and Statistical Manual of Mental Disorders, Fourth Edition (DSM-IV) diagnosis of schizophrenia based on a Structured Clinical Interview for DSM-IV (SCID)^35^, (2) age between 18 and 60 years, (3) sufficient competence in English language to understand testing procedures, (4) no clinically significant neurological disease as determined by medical history, (5) no history of serious head injury, (6) no evidence of substance or alcohol abuse in the month previous to testing, (7) no sedatives or benzodiazepines within 12 h of testing, (8) no history of intellectual disability or developmental disability, and (9) clinical stability. Selection criteria for community controls were: (1) age between 18 and 60 years, (2) sufficient competence in English language to understand testing procedures, (3) no clinically significant neurological disease as determined by medical history, (4) no psychotic disorder, bipolar disorder, or recurrent major depressive disorder according to SCID-I, (5) no schizotypal, avoidance, schizoid or paranoid personality disorder according to SCID-II, (6) no family history of psychotic disorders among first-degree relatives, (7) no history of substance or alcohol dependence and no substance or alcohol abuse in the month previous to testing, and (8) no sedatives or benzodiazepines within 12 h of testing.

Clinical symptoms of patients were assessed with the Scale for the Assessment of Negative Symptoms (SANS)^36^ and the Brief Psychiatric Rating Scale (BPRS)^37^. Diagnostic interviews, BPRS, and SANS were administered by trained diagnosticians. To characterize neurocognitive ability of participants, we administered the MATRICS Consensus Cognitive Battery (MCCB)^38,39^. The MCCB includes six different non-social cognitive domains (speed of processing, attention and vigilance, working memory, verbal learning, visual learning, and reasoning/problem solving).

### Measures

Five objective measures and one subjective measure of social cognition were administered. The objective measures include Facial Affect Identification^29^, Emotion in Biological Motion^29^, Self-Referential Memory^29,40^, Empathic accuracy^29,41^ and the Mayer–Salovey–Caruso Emotional Intelligence Test 2.0 (MSCEIT) Branch 4^42^, and the subjective measure was the Interpersonal Reactivity Index (IRI)^43^. As details of each measure are provided elsewhere^29,43^, we briefly describe each measure below.

In the Facial Affect Identification task, participants were asked to decide which emotional expressions a face conveyed on each trial. The primary dependent measure was percent accuracy.

For the Emotion in Biological Motion, participants were asked to decide which emotion (fear, anger, happiness, sadness or neutral) was described by the movement of a point-light walker stimulus. The primary dependent measure was percent accuracy.

For Self-Referential Memory task, participants first completed an encoding phase in which they decided whether a trait word described themselves (“self-referential” condition), whether the word indicated a desirable trait (“other” condition), and whether it was upper case. After a delay period, participants were presented one word at a time and asked to decide if the word was presented during the encoding phase. The primary dependent measure was an index of sensitivity (*d*’) for recognition of words.

We used two versions of an Empathic Accuracy task, and approximately half of the sample took the older version, and the other half took the newer version^29^. The key difference between the two versions was the diversity of individuals featured in the videos (i.e., targets), as the newer version was developed to include a broader range of age, racial and ethnic diversity. The dependent measure was the mean correlation across clips between the ratings of the targets on their own emotion and the participant’s ratings of the targets’ emotion. We did not find any performance difference between participants who received the older version and participants who received the newer version (see Supplemental material for details).

The MSCEIT Branch 4, Managing Emotion, assessed emotion regulation in oneself and one’s relationship with others using vignettes. Specifically, participants are presented with vignettes of various social situations along with the solution to cope with the emotions depicted in these vignettes. Participants are asked to indicate how effective each solution is using a scale ranging from 1 (very ineffective) to 5 (very effective).

Finally, the IRI was used as a measure of subjective social cognitive ability. The IRI, as a measure of empathy, consists of four subtests, each assessing a different aspect of empathy. The Fantasy subscale measures a tendency to transpose oneself into the feelings of a character in a movie or book. The Perspective Taking scale measures how a person will spontaneously adopt someone else’s point of view. The Empathic Concern Scale assesses feelings of sympathy or concern towards the other. The Personal Distress Scale measures feelings of personal distress in unpleasant interpersonal situations. We analyzed both the total IRI index and the four subscales separately.

In addition to measures of social cognition, we assessed functional capacity and community functioning of patients. Functional capacity was assessed using the University of California at San Diego Performance-Based Skills Assessment UPSA^44^; and the Maryland Assessment of Social Competence MASC^45^;. The UPSA consists of role-play simulation tasks that measure a participant’s ability to negotiate real-world tasks. As a measure of social skills (i.e., functional capacity on social domain), the MASC employs a role-play approach in which participants are responsible for taking the conversation forward in a series of common interpersonal problems. Four role play scenarios were videotaped and coded by trained raters who achieved a median interclass coefficient of 0.85 on a set of 10 videos that were derived from a separate sample. Community Functioning was assessed with the Role Functioning Scale RFS^46^;.

### Statistical analysis

First, to examine whether female and male patients with schizophrenia differ on demographic and clinical characteristics, we conducted a series of two-way ANOVA with group and sex as between-subject factors for age, personal education, and parental education, and one-way ANOVA for clinical characteristics. Second, to examine whether female and male schizophrenia patients show different levels of performance on objective and subjective social cognitive tasks, we conducted a series of two-way ANOVAs with sex and group as between-subject factors for all tasks except the Self-Referential Memory task. For the Self-Referential Memory task, we conducted a repeated measures ANOVA with condition as within-subject factor and group and sex as between-subject factors. Significance thresholds for the objective social cognitive tasks were set at *p* = 0.05 because each cognitive task is considered a separate task that assesses a distinct social cognitive domain. The subjective social cognitive measure, the IRI, includes four subscales and a total score; thus, significance thresholds for the subjective social cognitive task were set at *p* = 0.01 (0.05/5). All *p* values represent two-tailed tests. For these analyses, we also report effect size (i.e., partial eta square) along with statistics. The general rule of thumb regarding the magnitude of effect size for partial eta square is: 0.01 = small effect, 0.06 = medium effect, and 0.14 for large effect^47^.

Third, to examine whether sex moderates the associations between social cognition and functioning (i.e., functional capacity and community functioning) within the schizophrenia group, we conducted linear multiple regression analyses for objective social cognition and subjective social cognition separately. For objective social cognition, a social cognitive composite score was created by calculating the mean of the standardized objective social cognition variables using the mean and standard deviation of the control group. In the first block, the social cognitive composite score was entered, which allowed to compare the findings of this study to previous work on the relationship between social cognition in schizophrenia. Sex (dummy coded) was entered in the second block. In the third block, the interaction between sex and the social cognitive composite was entered. A significant interaction would indicate that sex moderated the relationship between social cognition and functioning in schizophrenia. A similar regression analysis was conducted for subjective social cognition using IRI total score, such that IRI total score was entered in the first block, followed by sex in the second block, and a sex by IRI total interaction in the third block. Significance thresholds represent two-tailed tests and were set at a *p* = 0.05.

### Reporting summary

Further information on research design is available in the Nature Research Reporting Summary linked to this article.

## Supplementary information

Supplementary information

Reporting Summary

## Data Availability

The dataset analyzed during the current study can be available upon request.

## Code availability

The code used for data analysis can be available upon request.
