# Supplementary material for: The effect of sex on social cognition and functioning in schizophrenia
Source: NPJ Schizophr. 2021 Dec 1;7:57. doi: 10.1038/s41537-021-00188-7 (PMC8636592; doi:10.1038/s41537-021-00188-7)
Supplement: Supplementary file 1 — Supplementary information [file 41537_2021_188_MOESM1_ESM.pdf]

Supplementary Material

Supplementary Method

Regarding the two versions of the Empathic Accuracy task, we did not find any significant difference between participants who were administered the newer version of the Empathic Accuracy task and participants who were administered the older version (patients, mean=.52 (SD=.17) and mean=.59 (SD=.17) for the newer and older versions, respectively; controls, mean=.61 (SD=.10) and mean=.70 (SD=.10) for the newer and older versions, respectively).

Supplementary Results

To better understand the sex difference on MASC and RFS total, we examined sex difference on subscales of MASC and RFS separately. For MASC, we found significant sex effects on all three subscales of MASC (MASC Verbal, $F_{(1,236)}=9.46$, $p<.01$, $\eta^2_p=.039$; MASC Nonverbal, $F_{(1,236)}=6.31$, $p<.05$, $\eta^2_p=.026$; MASC Effectiveness, $F_{(1,236)}=9.09$, $p<.01$, $\eta^2_p=.037$). We found a significant sex difference on RFS work ($F_{(1,331)}=7.62$, $p<.01$, $\eta^2_p=.023$) and RFS social functioning ($F_{(1,331)}=7.28$, $p<.01$, $\eta^2_p=.022$) and a marginally significant effect on RFS family functioning ($F_{(1,331)}=3.08$, $p=.08$, $\eta^2_p=.009$). A sex effect was not significant on RFS Independent living. Female patients with schizophrenia showed better work functioning, social functioning and family functioning.

Supplementary Table 1. Correlations between social cognition and functioning in the schizophrenia group

	Female			Male		
	UPSA total	MASC total	RFS total	UPSA total	MASC total	RFS total
Social Cognitive Composite Score	.394**	.283*	.325*	.639**	.242**	.257**
IRI total	-.03	.198	-.046	.017	.116	.054

* denotes p<.05 and ** denotes p<.01.

Abbreviations: UPSA, the University of California at San Diego Performance-based Assessment; MASC, the Maryland Assessment of Social Competence; RFS, the Role Functioning Scale

Supplementary Table 2. Performance on objective and subjective social cognitive tasks

	Patients		Controls		Statistics
	Female	Male	Female	Male	
**Objective Social Cognition**					
Facial Affect Recognition	.77 (.14)	.75 (.13)	.82 (.08)	.82 (.08)	Group $F_{(1,329)}$=20.14, p<.001, η^2_p=.058<br>Sex $F_{(1,329)}$=1.88, p=.17, η^2_p=.006, n=1303<br>Group by Sex $F_{(1,329)}$=.001, p=.98, η^2_p=.000, n=19617
Emotion in Biological Motion	.70 (.12)	.67 (.13)	.77 (.11)	.76 (.09)	Group $F_{(1,323)}$=21.76, p<.001, η^2_p=.063<br>Sex $F_{(1,323)}$=1.19, p=.27, η^2_p=.004, n=1957<br>Group by Sex $F_{(1,323)}$=.57, p=.44, η^2_p=.002, n=3919
Self-referential Memory					
Physical	.84 (.66)	.68 (.57)	.66 (.55)	.57 (.61)	Group $F_{(1,325)}$=1.98, p=.15, η^2_p=.006 n=1303<br>Sex $F_{(1,325)}$=2.82, p=.09, η^2_p=.009, n=867<br>Group by Sex $F_{(1,325)}$=.10, p=.75, η^2_p=.000, n=19617
Other	1.17 (.80)	1.07 (.69)	1.33 (.66)	1.21 (.56)	Condition $F_{(2,650)}$=227.04, p<.001, η^2_p=.411<br>Condition by Group $F_{(2,650)}$=21.08, p<.001, η^2_p=.061<br>Condition by Sex $F_{(2,650)}$=.46, p=.62, η^2_p=.001, n=7843
Self	1.32 (.81)	1.22 (.73)	1.73 (.67)	1.47 (.63)	Condition by Group by Sex $F_{(2,650)}$=1.41, p=.24, η^2_p=.004, n=2403
Empathic Accuracy	.59 (.17)	.55 (.15)	.66 (.12)	.67 (.09)	Group $F_{(1,316)}$=24.13, p<.001, η^2_p=.071<br>Sex $F_{(1,316)}$=.71, p=.40, η^2_p=.002, n=3919<br>Group by Sex $F_{(1,316)}$=2.15, p=.14, η^2_p=.007, n=1116
MSCEIT Branch 4	39.48 (11.76)	36.04 (11.79)	52.26 (8.04)	48.39 (10.30)	Group $F_{(1,326)}$=69.03, p<.001, η^2_p=.175<br>Sex $F_{(1,326)}$=5.83, p<.05, η^2_p=.018<br>Group by Sex $F_{(1,326)}$=.02, p=.88, η^2_p=.000, n=19617
**Subjective Social Cognition**					
IRI Fantasy	13.6 (5.2)	13.4 (5.0)	14.8 (5.3)	12.1 (4.8)	Group $F_{(1,329)}$=.00, p=.99, η^2_p=.00, n=19617<br>Sex $F_{(1,329)}$=4.46, p<.05, η^2_p=.013<br>Group by Sex $F_{(1,329)}$=3.25, p=.07, η^2_p=.010, n=779
IRI Empathic Concern	20.1 (4.8)	18.7 (4.7)	22.1 (3.3)	19.2 (4.2)	Group $F_{(1,329)}$=4.18, p<.05, η^2_p=.013<br>Sex $F_{(1,329)}$=11.43, p<.01, η^2_p=.034

					Group by Sex $F_{(1,329)}$=1.42, p=.23, η^2_p=.004, n=1957
IRI Perspective Taking	16.9 (4.7)	15.9 (4.8)	19.3 (5.2)	17.9 (4.3)	Group $F_{(1,329)}$=11.32, p<.01, η^2_p=.033<br>Sex $F_{(1,329)}$=3.32, p=.06, η^2_p=.010, n=779<br>Group by Sex $F_{(1,329)}$=.73, p=.78, η^2_p=.000, n=19617
IRI Personal Distress	13.3 (4.9)	11.7 (5.1)	7.5 (3.4)	7.3 (4.3)	Group $F_{(1,329)}$=61.78, p<.001, η^2_p=.158<br>Sex $F_{(1,329)}$=1.98, p=.16, η^2_p=.006, n=1303<br>Group by Sex $F_{(1,329)}$=1.20, p=.29, η^2_p=.003, n=2611
IRI Total	64 (13.96)	59.8 (12.1)	63.7 (11.0)	56.7 (11.4)	Group $F_{(1,329)}$=1.03, *p=.31*, η^2_p=.003, n=2611<br>Sex $F_{(1,329)}$=11.58, *p<.01*, η^2_p=.034<br>Group by Sex $F_{(1,329)}$=.77, p=.38, η^2_p=.002, n=3919

† Abbreviations: MSCEIT, the Mayer-Salovey-Caruso Emotional Intelligence Test 2.0; IRI, the Interpersonal Reactivity Index

†† Values are given as mean (standard deviation).
